# Erysipelas

**Published:** 1892-08-06

**Authors:** 


### The Practitioner's Mirror and Retrospect.

[*The Editor will be glad to receive offers of co-operation and contributions from members of the profession. All letters should be addressed to* The Editor, The Lodge, Porchester Square, London, W.]

### MEDICINE.

### ERYSIPELAS.

Two distinct methods of treatment have been recently advocated for erysipelas, viz., mechanical and antiseptic. Of these mechanical methods, one modification has received the practical approval of Professor Wolfer, of Vienna.* He says that he is perfectly well aware of the different attempts to check erysipelas by means of applying adhesive plaster, but his own experience had never been such as to convince him of the benefit or injury that would arise from its careful application till very recently. As to the mode of applying the adhesive plaster, where the parts are covered with hair, as the head, it is better to shave them before applying the plaster; in females, however, it is not always necessary to shave, as the hair can be parted and tied to the crown without hindering the application of the strips. Before any of the strips of plaster be removed, care should be taken that a new band of plaster be applied. This removal sometimes arises from the primary application being placed rather near the focus of radiation. If the adhesive be applied exactly it will be found that the progress of the rosy colour will be checked, and will rarely go beyond this artificial line of constriction. In exceptional cases this rule may not be exactly adhered to, but in these cases it will not extend beyond two or three fingers' breadth. If the plaster be not exactly applied, the redness is likely to extend beyond its limitations within a few hours. The author gives a résumé of his last sixteen cases treated in this way, and reviewing the favcurable results, remarks that no one can deny the influence of the adhesive in limiting the extension of the inflammatory focus which is typically displayed in the case of the face, where the fever continued on an average of four to six days, and then disappeared. It may further be noticed in this series that the mechanical treatment of erysipelas of the face gives a more favourable prognosis than it does in the under extremities. In the latter the inflammatory progress does not seem to be limited with the same precision as it can be in the face. In attempting a theoretical explanation of this, it is more reasonable to believe that in the lower extremities the streptococcus finds its way inward in a deeper channel, through the ligatured portion by the sheathes of the muscles, and escaping beyond to the surface, pursues its onward ravages in the cutaneous surface; in the face this opportunity is absent as the bones lie near the surface. During five years sixty cases have been treated in the Professor's clinic by this method; two died from sepsis acutissima, while forty-eight had the disease decidedly limited, and recovery assured.

An elastic band is used by Dr. Hermann Krael, under the belief that the bacilli are much hindered in their progress when they encounter the firmer portions of the skin. Dr. R. G. Hebb advocated the method of applying constriction so long ago as 1874-5 "when antiseptic surgery was only practised in out of the way places, and the phagocyte unknown." With this end in view he made use of an elastic band, encircling the head with it at the edge of the hair. The band must press firmly against the head, but the disease may then spread over the whole face without extending into the hair at all. The band must not be removed until the swelling and bluish-red colour have disappeared from the artificial border. While many patients find the band disagreeable, one, who had on a former occasion had the disease spread all over the head, declared that the tension was not to be compared with that produced by the disease itself.

Ichthyol has been largely used by Dr. Klein. He has, however, restricted his conclusions to observations made on severe cases of erysipelas, and are as follows: (1) Ichthyol undoubtedly exerts marked influence on the development of the micrococcus of erysipelas in the skin, which may be attributable either to the reducing action which this remedy exerts on the tissues, or to a direct action exerted on the pathogenic micro-organisms, or to both of these causes.

* *Medical Press and Circular*, vol. ciii., No. 20.

(2) Treatment by icthyol reduces the duration of erysipelas at least half. (3) Treatment need not be continued, as a rule, longer than three or four days. By this time the disease is usually cured. (4) Under the influence of ichthyol the disease follows a much more benign course. (Berl. Kim. Woch. Sept. 28th, 1891.)*

The results obtained by Dr. Klein led to the adoption of ichthyol in erysipelas by Dr. Radcliffe, whose experience with the drug had been very similar. He finds that the best plan of using it is to compound an ointment of equal parts of ichthyol and vaseline or lanoline, or if a weaker or thinner one is desired, equal parts of ichthyol, lanoline and water will be better where a large cutaneous surface is under treatment. He has this applied uniformly over every part of the erysipelatous inflammation, ears, eyelids and all, and repeats this at least twice daily. In three days, on washing it off with a little tepid water, and with or without a little lather of fine quality of soap, it will be found that the swelling has subsided, and the erysipelatous process has been arrested, except perhaps on the extreme borders. After three days it may be discontinued, and the paler parts dusted with flour, applying the ichthyol only to the outskirts, the irregular parts of the pinna of the ears and back of the neck, but perhaps a better plan is to continue this application till the sixth day, or until all traces of the disease externally have disappeared. No relapses have taken place in the author's experience, and convalescence was always satisfactory and without sequelæ.

Ichthyol collodion has been used by Sach and Schneider. A ten per cent. solution of ichthyol in collodion is painted over the affected area, but Schneider believes that collodion without ichthyol is just as efficacious as Sach's mixture.

One of the most interesting applications of the physiological action of a remedy to combat the local action of a microbal disease is the use of aconitine by Tison. At the outset of the disease he administers an emetic, after which he gives aconitine in the dose of $\frac{1}{200}$ of a grain every six hours. The drug must be pushed actively in order that its full physiological effect may be shown.

* *Ther. Gaz.*, Dec. 15th, 1891; *Ibid*, May 16th, 1892.

